# Highlights of 30^th^ Annual Conference of Association of Medical Physicists of India (AMPICON-2009)

**Published:** 2010

**Authors:** S. D. Sharma

**Affiliations:** Radiological Physics and Advisory Division, Bhabha Atomic Research Centre, CTCRS, Anushaktinagar, Mumbai - 400 094, Maharashtra, India. E-mail: sdsbarc@gmail.com

The 30^th^ annual conference of Association of Medical Physicists of India (AMPICON - 2009) was organized during November 22 - 25, 2009 at Katriya Hotel and Towers, Hyderabad which was jointly hosted by MNJ Institute of Oncology and Regional Cancer Centre (MNJIO and RCC), Hyderabad and AMPI Andhra Pradesh Chapter, Hyderabad. The theme of the conference was “Advances in Medical Physics and Technology for Conformal Dose Delivery in Cancer”. About 400 delegates from India and abroad participated in the conference.

Prof. T. Mandapal, Director, MNJIO and RCC, Hyderabad, presided over the inaugural function and released the Souvenir and Book of Abstracts of AMPICON-2009. In the presidential address, Prof. Mandapal highlighted the recent developments in the field of radiation oncology and emphasized the need and importance of medical physicist in the use of advanced technology for delivering high precision radiotherapy by means of recent treatment techniques such as intensity modulated radiotherapy (IMRT), image guided radiotherapy (IGRT) and stereotactic body radiotherapy (SBRT). AMPICON-2009 was inaugurated by Padmashree Prof. K. Subba Rao (an eminent radiologist), Chairman, Indo-American Cancer Hospital and Research Center, Hyderabad. While delivering the inaugural address, Prof. Subba Rao summarized the importance of ionizing radiation in diagnosis and treatment of various diseases with special emphasis on cancer. He stated the need of proper training and judicious use of the radiation technology to accrue maximum benefit for the mankind. Prof. Subba Rao requested the Medical Physics community of India to give equal importance to diagnostic radiology as like radiation oncology and initiate comprehensive periodic quality assurance (QA) program for diagnostic radiology equipment and imaging techniques, which will help in significant dose reduction to patients undergoing various radiological examinations. In his opinion, this service of medical physicist will be considered the best service to humanity as several radiological examinations are conducted per day.

Dr. Michael Gillin, Professor and Director of Medical Physics, University of Texas MD Anderson Cancer Center, Houston, Texas, USA presented the keynote address on the topic “Medical Physics in the Decade of the 2020's”. In his lecture, Prof. Gillin described in detail the past and present of the Medical Physics service in Radiation Oncology and futuristic aspects of further automation in dose delivery and dose verification. He also emphasized the superiority of Monte Carlo based treatment planning systems over model based treatment planning systems and predicted the routine use of Monte Carlo based systems in dose planning for both photon and electron beams in the coming decade.

One hundred and forty nine scientific papers, including Dr. Ramaiah Naidu Memorial Oration (RNMO), Dr. M. S. Aggarwal Memorial Oration (MSAMO), 36 invited, 34 oral and 77 poster papers were presented at the conference. Dr. Bhagwat Ahluwalia, Professor Emeritus, Department of Radiation Oncology and Department of Radiological Sciences, University of Oklahoma Health Sciences Center, Oklahoma City, Oklahoma, USA, delivered the eighteenth RNMO. The RNMO award is bestowed on an eminent personality who has a long working experience in the field of medical physics with a good track record of academics, research, and clinical practice. The title of his deliberation was “Image Guided Radiation Therapy: Technologies and Techniques”. During the talk Dr. Ahluwalia described in detail the recent technology of radiation oncology such as IMRT, IGRT and volumetric modulated arc therapy (VMAT). A good detail of available onboard imaging systems was also included in this lecture. He concluded: “Imaging or rapid imaging prior to and during the treatment is the way to assure the goals of conformity of the radiation dose to the target volume under treatment”.

Dr. A. R. Reddy, Former Director, Defence Research and Development Organization, Ministry of Defence, Government of India, Jodhpur delivered Dr. M. S. Aggarwal Memorial Oration. The topic of Dr. A. R. Reddy's MSAMO lecture was “Targeted radionuclide therapy: Need for medical physicist in nuclear medicine”. In his lecture, Dr. A. R. Reddy elaborated the roles and responsibilities of medical physicists in radiation oncology vis-à-vis nuclear medicine and advocated the necessity of dedicated medical physics service in nuclear medicine in India.

A preconference workshop on “Online Verification of IMRT/ IGRT” was also organized at MNJIO and RCC, Hyderabad on November 21, 2009. Scientific presentations and demonstrations related to QA in IMRT/ IGRT using electronic portal imaging device of the medical linear accelerator and commercially available independent dosimetry and QA systems were the main component of the workshop. Fifty Indian delegates and five overseas faculties participated in the workshop.

The scientific program of the conference was divided into 22 scientific sessions which covered the whole spectrum of medical radiation physics namely, Radiation Therapy Physics, Devices and Techniques; Medical Imaging Physics, Devices and Techniques; Radiation Dosimetry; Radiation Biology; Nuclear Medicine; Commissioning and QA of Imaging and Therapeutic Equipment; Monte Carlo and Empirical Dosimetry Methods in Medicine; Education and Training in Medical Physics; and Radiation Protection and Safety. Presentations on heavy ion therapy, recent developments in the technology of radiation medicine and methodology, and panel discussions on (i) Advances in Medical Physics Technology: Reach out to rural patients in affordable cost - Indian Scenario, and (ii) Education and Certification of Medical Physicists, were of special attractions to all. The oral and poster presentations were evaluated for the best Oral and Poster articles.

At the end of the conference, “Study of variation in dose calculation accuracy between kV Cone Beam CT (CBCT) and Conventional Planning CT” by K Venkatesan, Saraawathi Chitra, Pradeep Goswami, Amirtharaj from Galaxy Cancer Institute, Pushpanjali Crosslay Hospital, Ghaziabad, India and “Physical and Dosimetric Characteristic of High Definition Multileaf Collimator (HDMLC) for Image Guided Stereotactic Radiosurgery (IGSRS) and Intensity Modulated Radiotherapy (IMRT)” by Dayananda Sharma, Vaibhav Mhatre, Malhotra Heigrujam, Kaustav Talapatra, Suman Mallik from Department of Radiation Oncology, Kokilaben Dhirubhai Ambani Hospital and Medical Research Institute, Mumbai, India were declared the best Oral and Poster papers respectively.

Dr. Abhijeet Mandal, Medical Physicist, Department of Radiotherapy and Radiation Medicine, Banaras Hindu University, Varanasi was presented AMPI Young Investigator Award. This award was instituted by AMPI in 2005 to encourage young medical physicist who is doing commendable research and development work in clinics in the field of medical physics and is given to an AMPI Life Member who is less than 35 years of age and has a few good quality publications in refereed journals.

Scientific and trade exhibitions on external beam therapy, brachytherapy, medical imaging, different types of dosimetry systems, phantoms, computerized treatment planning systems, and treatment accessories were other attractions of AMPICON-2009. A number of recent technological equipments (Cyber Knife, RapidArc, VMAT, Tomotherapy, Gamma Knife, PET CT, Improved Telecobalt Machine, etc), on-line dosimeters, IMRT/ IGRT imaging and dose verification phantoms, patient immobilization devices and other related products were demonstrated in stalls arranged around the conference venue.

The conference deliberations and exhibitions were highly informative and advanced, which was useful for medical physicists, radiation oncologists, radiologists, radiobiologists, biomedical engineers, dosimetrists, and radiation technologists. The author was the Convener of the Scientific Program of AMPICON-2009.

